# Stakeholder governance and the CSR of banks: An analysis of an internal governance mechanism based on game theory

**DOI:** 10.3389/fpsyg.2022.918290

**Published:** 2022-07-25

**Authors:** Jiaji An, He Di, Meifang Yao

**Affiliations:** School of Business and Management, Jilin University, Changchun, China

**Keywords:** game theory, stakeholder governance, CSR of banks, agency theory, internal governance

## Abstract

Banks have an important social responsibility to serve the real economy and to maintain financial stability, and they also need to be responsible to borrowers and others. Against the backdrop of the COVID-19 pandemic affecting the global economy and increasing financial risks, it is particularly important for banks to assume social responsibilities. This study theoretically analyzed the outstanding applicability of stakeholder governance theory. Using a two-stage game method, the optimal pressure intensity of the social responsibility stakeholders was calculated, and the dynamic performance of shareholders was deduced. We found that the establishment of the social responsibility stakeholder governance mechanism will prompt the bank to fulfill its social responsibilities; rational social responsibility stakeholders will not lead to poor bank management due to excessive behavior; and shareholders with social responsibility can self-consciously choose the investment projects with lower negative externalities. The conclusions can be summarized as follows: The participation of stakeholder and the establishment of the social responsibility function of the board of directors can help promote a bank's social responsibility performance. This work studied the social responsibility of banks from the new perspective of stakeholder governance, expands the theoretical boundaries, and puts forward relevant suggestions to enhance the application value of this research.

## Introduction

Achieving a harmony that is evidenced by both economic efficiency and the social affordability of economic activity has become the goal pursued by the countries around the world today. As the hub of capital financing, the strategic orientation of the banking industry plays a pivotal role in reshaping the business ecological model of the common development of the business activities and environmental protection, which requires banks to undertake social responsibilities, that is, having the ability to choose and know how to fulfill the moral and legal responsibilities to protect the environment and promote a sustainable economic development while pursuing profit making and creating wealth. From the perspective of micro-operating entities, having an investment return is the code of conduct for the bank managers (Hassan et al., 2020). Does taking social responsibility pay off? In the long run, a bank's social responsibility will help to improve the customer satisfaction, enhance the brand value, and increase the revenue (Becchetti et al., 2020). However, in the short-run planning, benchmarking the bank performance against the Dominican 400 Social Responsibility Index even does not necessarily improve the performance (Kim, 2020). Banks need to adhere to the Equator Principles and incur additional costs, which will lead to lower financial performance (Tseng et al., 2017). Therefore, compliance with social responsibility may be more of a signaling behavior for the banks (Belasri et al., 2020). In this case, how can the banking industry fulfill its social responsibilities? Under the ambiguous situation of profit creation, it is clear that the corporate governance played the leading role for banks to fulfill their social responsibilities, because of the non-profit-seeking nature of CSR. The practices of the banking industry in Italy, Nigeria, and other countries show that the external management and the internal control are equally important, and the participation of multiple stakeholders to improve the corporate governance can promote the banking industry to fulfill its social responsibilities (Foglia and Angelini, 2019; Umar and Musa, 2020). To analyze the internal governance mechanism of banks, a method to improve the social responsibility level of banks needs to be obtained, and the application of the boundaries of the corporate governance theory and the concept of stakeholder governance need to be expanded. There are several main research questions that are discussed in this study: Do social responsibility stakeholders need to be included in the corporate governance structure? Can a shareholder's sense of CSR play a role in the social responsibility of banks? To answer these questions, we build game models and mathematically calculate the dynamics of the game players. Some conclusions about the positive effects of stakeholder governance and the attributes of shareholder social responsibility may be able to serve as references for the related research.

## Literature review

### The necessity of stakeholders' participation in corporate governance of commercial banks

The basic principles and governance framework of the corporate governance structure of the banks have the same characteristics as the general corporate governance structure as follows: The scientific decision-making needs to be realized. However, as a financial enterprise, the corporate governance structure of commercial banks has great particularity. Harkin et al. (2019) pointed out that the standard agency model used to study the general enterprises is not suitable for the banks because the goal that the commercial banks have of achieving corporate governance is not only to protect the interests of investors but also to reduce the market system risks and to maintain the stability of the financial system. The effective corporate governance is the foundation for gaining and maintaining public trust and confidence in the banking system, which is the key to the sound operation of the banking industry and the entire economic system. The commercial banks are different from general enterprises in terms of the contract products and capital structure, which determines the particularity of bank corporate governance in the aspects discussed in the following.

First, the degree of information asymmetry in the banking industry is relatively high (Asongu and Biekpe, 2018; Tran et al., 2019; Tessema, 2020). Due to the non-exclusive nature of information circulation, and financial products, the complexity of the bank principal–agent relationship and the non-marketization and non-standardization of bank assets (mainly loans) transactions, the bank asset transactions are not transparent. For example, people do not have immediate information on loan quality, and banks can quickly change the risk structure of their assets to hide problems. This non-transparency not only makes it very difficult for shareholders to supervise management but also weakens the pressure on the internal management from the control competition mechanism in the product market and in the capital market. Therefore, the restraint mechanism from the product market cannot play a fundamental role in the external governance mechanism of commercial banks (Zhang and Wu, 2020; Klein et al., 2021).

Second, strong regulation would limit the effect of market power and equity constraints on banks (Repullo, 2018; Calzolari et al., 2019; Grunewald, 2021). In view of the strong contagion effect of bank failures, governments have generally established safety nets to protect banks. The existence of the safety net has weakened the market constraints on banks to a certain extent, thus further highlighting the importance of the internal governance in banks. At the same time, to maintain the financial stability or to achieve other regulatory goals, governments tend to limit the concentration of ownership and control the ownership of banks, thereby hindering the competition for power. The constraints and interests in the banking business limit the important role of external competition on the corporate governance mechanism of banks. Levine (2004) believed that when the government is too involved in management activities, the corporate governance structure of commercial banks changes.

Third, the particularity of the capital structure of commercial banks further weakens the supervision of external claims (McDonald and Rundle, 2008; Du and Palia, 2018). The proportion of the bank's own capital is very low (according to the Basel Accord, the capital adequacy ratio of commercial banks is 8% to meet the requirements), which provides bank owners an incentive to invest in high-risk activities, which, in turn, harms depositors who receive only a fixed return (Dewatripoint and Tirole, 2004). Coupled with the fact that many scattered depositors have neither the motivation nor the ability to review and search for information or to intervene in the bank management, the role of creditor right constraints in the bank's governance structure is minimal (Du et al., 2018). Therefore, the lack of supervision of external creditor rights is an important particularity of the governance structure of commercial banks.

From the above analysis, it can be seen that the particularity of corporate governance structure in banks determines that the debt constraints and the product market constraints cannot play a fundamental role in the external governance and are not conducive to the formation of a supervision mechanism through which the banks to fulfill their social responsibilities. The profit-seeking nature of the banks determines that they will inevitably disregard the environment and ecological benefits during their operation. The market nature of banks determines that the implementation of bank ownership must rely on a standardized market mechanism. This requires the banks to not only need a complete set of internal governance mechanisms but also need a series of external governance mechanisms that function through the securities market, deposit and loan market, and manager market with the participation of social supervision forces and banking self-regulatory organizations (Occhino, 2017). The corporate governance of banks should adhere to the principle of “shared governance by stakeholders.” Through the joint participation of stakeholders including shareholders, employees, creditors, customers, and suppliers, the effective corporate governance can be formed, and the problem of information asymmetry in the original principal–agent relationship can be improved. At the same time, this will also prompt the banks to take the interests of their stakeholders more into account when making management decisions and to earnestly undertake the social responsibilities.

### Stakeholder governance: Connotation and structure

#### Connotation of stakeholder governance

Stakeholders are all of the individuals or groups that have contributed dedicated assets to an enterprise and who are already in venture capital status as a *fait accompli* (Razums and Laguna, 2018; Seibert et al., 2021). Given the differing or even contradictory goals, choices, and needs of the different stakeholder groups, the researchers usually divide them into two categories, primary and secondary stakeholders (Bridoux et al., 2016; Singh et al., 2020). Among them, the main stakeholders refer to those who are essential to the survival of the company itself or who have some kind of formal contract with the company (owners, shareholders, employees, customers, suppliers, etc.). The secondary stakeholders mainly refer to the social and political figures based on the corporate reputation (government, environmentalist organizations, etc.). Furthermore, Yang et al. (2018) extended stakeholders to the environmental sustainability.

Under the framework of corporate governance theory, stakeholder theory has two values, normative (ethics/morality) and instrumental (profit/increasing wealth) (Marques et al., 2018) values. Among them, the normative value stems from the idea of a social entity in the second half of the 19th century. This concept views corporations as public associations that are organized through politics and law and as social entities with public obligations and who pursue collective goals (Yazliuk et al., 2017). Under the guiding framework of the human rights and basic ethical values, the value of a company is not established just because it creates personal wealth, but because it respects the personal dignity, improves overall welfare, and helps the society gain a stronger sense of community (Sahut et al., 2019). The instrumentality of stakeholder theory is reflected in the pluralist model of the corporate governance (Wustenberg, 2019). Similar to social entity theory, the pluralist model also holds that the companies should serve the multiple interests of stakeholders rather than the shareholders. The difference is that it takes stakeholder values as an effective means to improve efficiency, profit, and competitiveness. The stakeholders should be involved in corporate decision-making because they make specific investments and take risks while also increasing the corporate efficiency (Rendtorff, 2018; Unterhitzenberger et al., 2020).

Due to the differences in the research perspectives of stakeholder theory, the current academic views on stakeholder governance are divided into the view of common stakeholder governance and the view of key stakeholder governance. The former's governance structure and mechanism are based on the belief that “all stakeholders” will lead to delayed decision making and stalemates in different opinions due to the decentralized control that will seriously affect the company's operational efficiency (Ma et al., 2019). It can also lead to the danger of publicizing the company, putting it in a situation where no one can really play a governance role, that is, full responsibility equals no responsibility (Harrison and Wicks, 2019). The key stakeholder governance concept also advocates collaborative governance but emphasizes that the basis of participation in governance lies in the key interests. Therefore, only those key stakeholders can participate in corporate governance. These people include shareholders who provide irreplaceable resources for the company's survival and core employees who take major risks in the company's operations (Tuapawa, 2017; Hristov and Appolloni, 2021; Restrepo-Olarte and Cogollo-Florez, 2021). This study prefers to rely on the key stakeholder governance perspectives.

Whether from a normative or an instrumental viewpoint, stakeholder participation in governance has become an important part of corporate governance and has been widely recognized both in theory and in practice. For example, the “Principles of Corporate Governance Structure” issued by the Organization for Economic Cooperation and Development (OECD) pointed out that the framework of the corporate governance structure should recognize the legal rights of stakeholders and encourage companies and stakeholders to actively cooperate to create wealth, job opportunities, and maintain their own financial health. As a major revision of the traditional shareholder, the sovereignty governance, and manager-led governance, stakeholder governance reflects the transformation of the corporate governance model from a single incentive to multiple incentives (Ortas et al., 2019). This theory reflects that the enterprise is a contractual connector that creates value jointly by including various participants and emphasizing the common governance of the enterprise by various stakeholders and is more conducive to mobilizing the enthusiasm of all stakeholders (Gurzawska, 2020).

#### Structure of stakeholder governance

In practice, embedding the interests of uninvested stakeholders into corporate governance is undoubtedly an extremely challenging task. The main difficulty is that the legal perspectives of corporate social responsibility (CSR) and management's fiduciary responsibility do not seem to be compatible. The latter clearly states that the managers must serve the interests of the company and its shareholders. Friedman advocated that “corporate social responsibility is to increase profits” is the best interpretation of this point of view. Therefore, although Tirole (2001) proposed the concept of “stakeholder society,” he also had to admit that so far, there is no complete set of governance mechanisms through which this concept can be implemented. The recent studies on stakeholder governance have only linked the corporate governance with the social and the environmental responsibilities, and few studies have analyzed how to integrate the interests of different stakeholders into corporate decision-making and management processes (Feshchur et al., 2018; Fernandez Torres and Villena Alarcon, 2021).

From the existing research, the content of stakeholder governance should include good corporate governance (protecting shareholders' interests), solid stakeholder relationships (protecting the interests of other stakeholders such as employees and communities), and environmental efficiency (protecting environmental interests) (Zappi, 2007). The stakeholder participation in corporate control requires a diverse board control structure (Dato et al., 2019). From an organizational theory perspective, the appropriate structure depends on the factors such as technology, size, environmental changes, strategy, interest groups, and culture. Specific to the governance structure of stakeholders, Amis et al. (2020) believed that research can be carried out from the three perspectives discussed in the following.

The first is the perspective of the social responsibility of the board of directors. This is related to a key issue in corporate governance—board structure. Board structure refers to the type and number of committees established by the board, committee membership, information flow between committees, etc. (Bolibok, 2021). Compensation and personnel committees, audit committees, and shareholder relations committees help protect the shareholder rights, as evidenced by numerous studies on agency theory (McMullen, 1996; Andronikidis et al., 2020). There is evidence that the purpose-built committees are effective in addressing the related issues. For example, Clune et al. (2014) found that large number of the USA-listed companies with nominating committees have more market and performance advantages than those without such committees. Linck et al. (2008) pointed out that adjusting the board structure to adapt to the requirements of a sustainable development can better ensure the formation of the company's sustainable strategy (including properly handling the relationship with the various stakeholders) and can improve the implementation of the strategy quality and depth. Jackling and Johl (2009) examined the impact of considering the interests of multiple stakeholders and incorporating social responsibility or sustainable development issues into the board structure on company performance. The study found that when the board clearly fulfilled its social responsibilities, stakeholders were able to participate in corporate governance more.

The second is board diversity. Within the framework of corporate governance, the board diversity can be expressed as differences in age, gender, ethnicity, culture, religion, region, professional background, knowledge, skills, expertise, and business and industry experience among the directors (Bear et al., 2010). From an ethical point of view, the stakeholder representatives should have a fair way of expressing their demands. It is the responsibility of the company to reflect the diversity of interests in its board of directors. According to the resource-dependence theory, the diversity of the demographic characteristics of the board of directors increases the external resource access of the organization, which is beneficial for the company to build relationships with different stakeholder groups. This leads to a deeper understanding of the market, fosters innovation, and solves the problems more effectively, thereby increasing the efficiency of the company's operations. For example, the participation of employee representatives on the supervisory board can improve the efficiency and market value of the company (Erhardt et al., 2003; Harjoto et al., 2015). Women are more sensitive to social responsibility issues, and the participation of female directors is more beneficial for the organizations to fulfill their social responsibilities (Sila et al., 2016). The diversity management has now become an important indicator of corporate social responsibility and is used in the social responsibility rating systems such as KLD Indexes and the FTSE4Good Index.

The third is stakeholder engagement. According to the stakeholder–agent theory put forward by Hillman and Dalziel (2003), the managers should act as the agents of the stakeholders. Since the stakeholder expectations regarding social responsibility are not static, to obtain accurate information about these expectations, the companies must develop corresponding strategies and establish a two-way dialogue mechanism between the company and its stakeholders (Agle et al., 2008). Research shows that through the strategic dialogue mechanism, explicitly including stakeholders in the CSR strategy formulation process will improve the quality of the CSR strategy (O'Riordan and Fairbrasss, 2008). Those companies that rank at the top in terms of sustainability benefit in large part from governance mechanisms that include stakeholders (Ricart et al., 2005).

## Methodology

Game analysis is the use of mathematical methods to study whether two parties with conflicts of interest have their own optimal strategies that they can implement to win against the other party in competitive activities and studies how to formulate these strategies. Game analysis has been deeply used in psychology, environmental science, transportation, management, sociology, library information and archives, and other fields (Aumann and Maschler, 1985; Hobbs et al., 2000). At present, in the corporate governance and social responsibility research field, game analysis is gradually becoming a new and effective method (Kruitwagen et al., 2017). Based on the traditional agency theory, the game has only two players, namely, shareholders and managers. However, this study introduces stakeholders into the bank's corporate governance system; therefore, there are three players, and a two-stage game model is formed. These include the games between stakeholders and shareholders and that between the shareholders and managers. The premise of the game assumes that the players are all self-interested rational people. Other game settings and specific models will be detailed in the game deduction process.

## Stakeholder governance and banks' social responsibility: Game analysis under the principal–agent framework

Corporate social responsibility refers to treating the stakeholders ethically or in a responsible manner (Cho et al., 2019). Jizi et al. (2014) designed steps for the development of the banking industry and the fulfillment of corporate social responsibility. With reference to the above, we designed the research objectives of this study as follows: (a) Identify different stakeholder groups; (b) Analyze the expectations of different stakeholders; (c) Design products and strategies that meet stakeholder expectations; (d) Integrate the strategy into the overall development strategy of the bank; (e) Perform ongoing assessment of the changes in social systems and in social needs. Jizi et al. (2014) even defined CSR as a multi-stakeholder-oriented corporate strategy and believes that it is related to the bank's global strategy, rather than an additional activity beyond traditional functions. Then, as an institutional arrangement that incorporates the consideration of the interests of stakeholders into the corporate governance structure, can stakeholder governance enable companies to fulfill their social responsibilities? To solve this problem, this study will conduct a game analysis of corporate governance and the ability of banks to fulfill their social responsibilities with the participation of multiple subjects.

### Establishment of game payoff matrix

Suppose the bank has a group of shareholders “*S*” and is managed by a manager “*M*.” Also, “*A*” represents the CSR activists among the stakeholders (CSR group representative); “*j*” represents the players in the game. Each participant is risk neutral and aims to maximize their expected return. *j* ∈ {*M, S, A*} represents the payoff function for player *j*, and *k* ∈ {*M, S, A*} represents the payoff function of player *j* when the item with preference *k* is executed. The bank has *N* + 1 projects to be decided, which are expressed as *i* = 0, 1, ..., *N*, *N* ≥ 5. Suppose that the bank has four special projects: item *i* = 0 is an existing item, and it pays 0 to shareholders (*S*), managers (*M*), and CSR activists (*A*). The other three projects provide *S*, *M*, and *A* with the corresponding highest returns, respectively. Let *i*_*j*_ be the participant *j*'s preference item assuming that the three participants' preference project are different, that is, there is a conflict of interest among them. Manager, *M*, achieves the highest personal benefit *B*^*M*^ > 0 when he performs his preferred item *i*_*M*_. When the manager performs a project preferred by the other participants, then *B*^*j*^, 0 < *B*^*j*^ < *B*^*M*^. The later players of the game are *j* = *S, A*. When executing item *i*_*S*_ preferred by *S*, the maximum benefit that shareholders can obtain is Π^*S*^ > 0, 0 < Π^*j*^ < Π^*S*^, *j* = *M, A*. Under the assumption of a rational person, both projects *i*_*M*_ and *i*_*S*_ will cause negative externalities to stakeholders. Furthermore, *A* represents the stakeholders, so the negative externality can be interpreted as *A*'s payment Ω^*j*^. Let Ω^*A*^ = 0 be the minimum value paid by *A*, Ω^*j*^ > Ω^*A*^ ≥ 0, *j* = *M, S*. When Ω^*A*^ = 0, the existing project is no different from the project supported by *A*, and *A* will not put pressure the execution of the existing project, which is *A*'s preference. Considering that shareholders may have a sense of social responsibility, we introduced γ as the social responsibility coefficient; γ_*S*_ represents the social responsibility of *S*, γ_*s*_ ∈ [0, 1]. Therefore, the maximum benefit of *S* from project, *i*, is revised to be $\Pi^{j}\text{-}\gamma_{S}\Omega^{j}$ by taking into account the cost of social responsibility, *j* = *M, S*. Based on the above, the description of the main variables designed by the game is shown in Table 1. The payoff matrix of the three game participants is shown in Table 2.

**Table 1:** Description of the main variables of the game.

**Symbol**	**Meaning**	**Ranges**
*S*	Shareholders of the bank	-
*M*	Bank manager	-
*A*	Socially responsible stakeholder representatives	-
*j*	Players of the game	*j* ∈ {*M, S, A*}
*k*	Payoff function of player	*k* ∈ {*M, S, A*}
*i*	Player's preference item	*i* ∈ {*M, S, A*}
Π^*j*^	Shareholders' own benefits when implementing project *j*	Π^*S*^ ≥ Π^*j*^ > 0*j* ∈ {*M, S, A*}
*B* ^*j*^	Manager's own benefits when implementing project, *j*	*B*^*M*^ ≥ *B*^*j*^ > 0*j* ∈ {*M, S, A*}
Ω^*j*^	Stakeholder representatives' payment when implementing project, *j*	Ω^*j*^ ≥ Ω^*A*^ ≥ 0*j* ∈ {*M, S, A*}
γ_*S*_	Social responsibility coefficient of shareholders	γ_*s*_ ∈ [0, 1]
*e*_*M*_	Manager got related information with probability	*e*_*M*_ ∈ [0, 1]
*e*_*S*_	Shareholders got related information with probability	*e*_*S*_ ∈ [0, 1]
*e*_*S*_ ∈ [0, 1]	Probability of occurrence of stakeholder representatives	*p* ∈ [0, 1]
λ	Pressure intensity of stakeholder representatives	λ ∈ [0, 1]
λ ∈ [0, 1]	Loss of *A*'s own interests caused by *A*'s pressures	-
*C*_*j*_	Loss of revenue due to selection of CSR projects	*j* = *M, S*

**Table 2:** Payoff incurred by the three-participant project.

**Participant Payoff**	**Project Payoff**			
	**0**	***i*_*M*_**	***i*_*S*_**	***i*_*A*_**
M	0	*B* ^*M*^	*B* ^*S*^	*B* ^*A*^
S	0	$\Pi^{M}\text{-}\gamma_{S}\Omega^{M}$	$\Pi^{S}\text{-}\gamma_{S}\Omega^{S}$	Π^*A*^
A	0	-Ω^*M*^	-Ω^*M*^	0

The loss of all participants in the game is the negative number of project payoff.

### Game deduction

#### Game assumptions

Dynamic games unfold in time series, *t* ∈ [1, 6]. When *t* = 1, the manager and shareholders begin to collect information to understand the level of payoff from all programs to all participants. The manager or shareholders obtain such information with the probability *e*_*M*_ or *e*_*S*_. To highlight the manager's role in the management structure, it is assumed that if *M* does not obtain the information, then *S* cannot obtain it either. This is also more in line with the actual bank operation. When *t* = 2, *M* announces the project that he wants to execute. When *t* = 3, *S* may veto *M*'s decision (assuming there is no cost to veto) and announce another project. When *t* = 4, *S*'s selected projects are executed. When *t* = 5, the CSR activists may put pressure on bank. When *t* = 6, the shareholders may decide to back down due to pressure. Since this study focuses on the impact of stakeholder participation in the principal–agent relationship, only the last two periods are discussed.

We assumed that at *t* = 5, where the probability of occurrence of *A* is *p*, 0 < *p* < 1. After observing the proposed project, *A* may put pressure on the bank due to negative externalities, such as boycotts or strikes. It is assumed that these pressures can be translated into a reduction in shareholder profits by the ratio λ (0 < λ ≤ 1). Also, *c*_*A*_(λ) is the loss of *A*'s own interests caused by *A*'s pressures on the bank, *c*_*A*_(0) = 0. Here, λ can be interpreted as the stress level.

At *t* = 6, *S* may yield to *A*'s pressure and instruct *M* to terminate the project being performed (*t* = 4) and to instead perform *A*'s preferred project. This results in the cost *C*_*j*_ ≥ 0, *j* = *M, S*, $C_{S}<\Pi^{A}$ (shareholders profit does not matter). To focus on the most preferred project, *i*_*j*_, of the three game players, *j* ∈ {*M, S, A*}. We set Ω^-*A*^ to represent the negative externality that *A* has to undertake in projects other than *i*_*A*_.

*Assumption 1*. $c_{A}\left(1\right)\leq \max\left\{\Omega^{\text{-}A}\right\}$ shows that the cost of pressure is no greater than the negative externalities that *A* bears. As such, *A* will put pressure on all projects except for the *i*_*A*_ he prefers.

*Assumption 2*. When considering the possibility of being rejected by *A*, the expected benefit (1-*p*)*B*^*M*^ + *p*(*B*^*A*^ − *C*^*M*^) obtained by *M* from its preferred project, *i*_*M*_, is the largest. In the same way, $\left(1\text{-}p\right)\left(\Omega^{s}-\gamma_{s}\Omega^{s}\right)+p\left(\Omega^{A}-C_{S}\right)$ is the maximum profit that *S* can obtain.

*Assumption 3*. *A* has enough power to apply pressure.

#### Model solving

We used reverse induction to solve the model. For ease of interpretation, the shareholders are rational about whether or not to compromise. The CSR activists do not directly influence the manager judgments, but they can change manager's decisions by putting pressure on the shareholders. Therefore, there are two sets of game relationships between the three, namely, the game between *A* and *S*, and the game between *S* and *M*.

1. The game between the CSR activists and shareholders.

At *t* = 6, if *A* has exerted pressure, *S* must decide whether to compromise, and *S* will compromise under the pressure from *A* if and only if the cost of compromising is less than the cost of insisting on executing project *i*_*j*_. From this, we obtain Condition 1.

*Condition 1*


(1) $$\begin{array}{l} \left(\Pi^{A}\text{-}C_{S}\right)\geq \left(1\text{-}\lambda \right)\left(\Pi^{j}\text{-}\gamma_{S}\Omega^{j}\right),orequivalently\\ \lambda \geq 1-\left(\Pi^{A}\text{-}C_{S}\right)/\left(\Pi^{j}\text{-}\gamma_{S}\Omega^{j}\right)\equiv \alpha_{1}^{j} \end{array}$$

“≡” represents the identity marker for condition 1, which is the same as below. Under this condition, the shareholders will yield.

We noted that since ≡, this condition requires $\Pi^{A}\text{-}C_{S}>0$ and $\Pi^{j}\text{-}\gamma_{S}\Omega^{j}>0$. We assumed that the cost, *C*_*S*_, of *S* to compromise is lower than Π^*A*^ to avoid a situation where the shareholders never compromise. We also assumed that after taking the social responsibilities into account, the benefits incurred by *S* from its most preferred projects are still large enough. In other words, shareholders still find their favorite projects to be the most attractive in some cases. As described above, at *t* = 5, if and only if the cost of pressure is no greater than the negative externalities that *A* bears, *A* will put pressures on *S*. From this, we obtain Condition 2.

*Condition 2*


(2) $$\begin{array}{l} c_{A}\left(\lambda \right)<\Omega^{j},orequivalently\\ \lambda <c_{A}^{-1}\left(\Omega^{j}\right)\equiv a_{2}^{j} \end{array}$$

Under this condition, *A* will put pressure on project *i*_*j*_.

Combining Conditions 1 and 2, we obtain the first game steady state (ESS). Only when *A* is willing to apply pressure and *S* will compromise with the pressure, the project that is preferred by *A* will be executed at *t* = 6. That is, Equations (1) and (2) are established at the same time.


(3) $$\begin{array}{l} c_{A}^{-1}\left(\Omega^{j}\right)>\lambda \geq 1-\left(\Pi^{A}\text{-}C_{S}\right)/\left(\Pi^{j}\text{-}\gamma_{S}\Omega^{j}\right) \end{array}$$

2. The game between the shareholders and manager.

When *t* = 2 and *t* = 3, the shareholders must decide whether to overrule the manager's decision. As mentioned earlier, the shareholders can only obtain information if the managers also obtained information. According to Assumption 2, in any case, after considering various costs, the participants will still choose their preferred project. Therefore, the shareholders can only approve the manager's project (*i*_*M*_) if the manager has collected the information and the shareholders have not. However, if the shareholders obtained the information, then according to Assumption 2, *S* will choose their preferred project (*i*_*S*_) and will reject the project (*i*_*S*_).

At *t* = 1, the shareholders and manager are struggling to gather information about the game. Their probability of acquiring information is *e*_*S*_ and *e*_*M*_, which is related to their effort level, and the cost they pay for collecting information is positioned as $e_{j}^{2}/2$, *j* = *M, S*. Suppose *A* exerted λ degree of pressure, $\lambda^{Ij^{*}}=a_{1}^{j}=1-\left(\Pi^{A}\text{-}C_{S}\right)/\left(\Pi^{j}\text{-}\gamma_{S}\Omega^{j}\right)$. That is, the project to be executed finally will be *i*_*A*_, the project preferred by *A*, according to Condition 1. At this point, the manager's expected utility expression is expressed as


(4) $$\begin{array}{l} U_{M}^{I}=e_{M}e_{S}\left[\left(1-p\right)B^{S}+p\left(B^{A}-C_{M}\right)\right]\\ +e_{M}\left(1-e_{S}\right)\left[\left(1-p\right)B^{M}+p\left(B^{A}-C_{M}\right)\right]-e_{M}^{2}/2 \end{array}$$

The shareholder's expected utility expression is expressed as


(5) $$\begin{array}{l} U_{S}^{I}=e_{M}e_{S}\left[\left(1-p\right)\left(\Pi^{S}-\gamma_{S}\Omega^{S}\right)+p\left(\Pi^{A}-C_{S}\right)\right]\\ +e_{M}\left(1-e_{S}\right)\left[\left(1-p\right)\left(\Pi^{M}-C_{S}\right)\right]-e_{S}^{2}/2 \end{array}$$

The establishment of the expected utility above is based on the following three probability distributions: First, under the probability of *e*_*M*_*e*_*S*_, both manager and shareholders are informed, and based on Assumption 2, the shareholders will overrule the manager's decision and require the implementation of *i*_*S*_. Second, under the probability of *e*_*M*_(1 − *e*_*S*_), only the manager has access to the information, and the shareholders' optimal choice is to agree to perform *i*_*M*_. Third, under the probability of (1 − *e*_*M*_)(1 − *e*_*S*_), the shareholders and manager have no information and can only execute existing projects, and the gain is zero.

Through calculations, we obtained the respective effort levels $e_{M}^{I*}$ and $e_{S}^{I*}$ under the condition of maximizing the expected return ($U_{M}^{I}$ and $U_{S}^{I}$) of the managers and shareholders.


(6) $$\begin{array}{l} e_{M}^{I*}=\frac{\left(1-p\right)B^{M}+p\left(B^{A}-C_{M}\right)}{1-{\left(1-p\right)}^{2}\left[B^{S}-B^{M}\right]X} \end{array}$$

(7) $$\begin{array}{l} e_{S}^{I*}=\frac{\left[\left(1-p\right)B^{M}+p\left(B^{A}-C_{M}\right)\right]\left(1-p\right)X}{1-{\left(1-p\right)}^{2}\left[B^{S}-B^{M}\right]X} \end{array}$$

(8) $$\begin{array}{l} X=\Pi^{S}-\gamma_{S}\Omega^{S}-\left(\Pi^{M}-\gamma_{S}\Omega^{M}\right) \end{array}$$

As before, the manager is the forerunner of information gathering and would consider all possible game probabilities; as such, the manager's optimal response function can be constructed using the formula expressed as follows:


(9) $$\begin{array}{l} e_{M}^{*}=e_{S}^{*}\left(1-p\right)\left(B^{S}-B^{M}\right)+\left(1-p\right)B^{M}+p\left(B^{A}-C_{M}\right) \end{array}$$

The construction principle of the formula expressed in Equation (9) has three probability distributions. First, the CSR activists did not appear, the shareholders obtained the information to negate the manager's choice, and the bank executes *i*_*S*_. Second, the CSR activists did not appear, the shareholders were not informed, and the bank implemented the manager's decision, *i*_*M*_. Third, the CSR activists emerged, and under pressure, the banks opted to implement, *i*_*A*_.

The shareholders are latecomers to information gathering, so their response function varies with the manager's response function, $e_{M}^{I*}$. Through the division operation of formulas expressed in Equations (6) and (7), we can determine the optimal ratio of the efforts of shareholders and managers.


(10) $$\begin{array}{l} e_{S}^{I*}/e_{M}^{I*}=\left(1-p\right)X \end{array}$$

Formula expressed in Equation (11) can be obtained by converting formulas expressed in Equations (8) and (10), that is, the best shareholder effort level.


(11) $$\begin{array}{l} e_{S}^{*}=e_{M}^{*}\left(1-p\right)\left[\left(\Pi^{S}-\Pi^{M}\right)-\gamma_{S}\left(\Omega^{S}-\Omega^{M}\right)\right] \end{array}$$

### Analysis of game results

After obtaining the steady state of the game and optimal solutions, the results need to be analyzed. The first is the game result, which is for the CSR activists and shareholders.

#### Lemma 1

Through the game, we obtained a stable CSR activist pressure intensity λ region: $c_{A}^{-1}\left(\Omega^{j}\right)>\lambda \geq 1-\left(\Pi^{A}\text{-}C_{S}\right)/\left(\Pi^{j}\text{-}\gamma_{S}\Omega^{j}\right)$. Since *c*_*A*_(λ) is an increasing function of λ, it is considered that when *A* increases the pressing force, the cost it pays will also increase, so the optimal pressing force is the minimum value that *A* needs to achieve. $\lambda^{j^{*}}=\left(\Pi^{A}\text{-}C_{S}\right)/\left(\Pi^{j}\text{-}\gamma_{S}\Omega^{j}\right)\in \left(0,1\right]$ is the best pressure that can be applied by CSR activists according to this study. In this case, the shareholders will choose the projects preferred by CSR activists, resulting in the least negative externalities, and the costs to be paid by CSR activists are also the least.

The second results are for the game between the shareholders and the manager. We analyzed Equation (9) first. As mentioned earlier, *B*^*M*^ is the maximum value of *B*^*j*^, so *B*^*S*^ − *B*^*M*^ < 0. This means that *e*_*M*_ decreases as *e*_*S*_ increases. Equation (11) should be reanalyzed since the meaning of $\left[\left(\Pi^{S}-\Pi^{M}\right)-\gamma_{S}\left(\Omega^{S}-\Omega^{M}\right)\right]$ cannot be determined, the effect of *e*_*M*_ on *e*_*S*_ is difficult to determine.

#### Lemma 2

If the shareholders have no sense of social responsibility, γ_*S*_ = 0, then the effect of *e*_*M*_ on *e*_*S*_ is obviously positive. In this case, the manager will first increase their effort level, *e*_*M*_, and then the non-socially responsible shareholders will also increase their effort level, *e*_*S*_. According to the formula used in Equation (9), it can be seen that this promotion will reduce the manager's effort level, which, in turn, affects the shareholders' effort level, and so on. Due to the settings of some of the parameters, the loop will stop at the equilibrium point. However, as mentioned above, when *e*_*M*_ and *e*_*S*_ reach a certain equilibrium, due to the absolute control of shareholders over the bank, it is very likely that the projects selected by shareholders will be executed. Therefore, if the shareholders of the bank are all socially irresponsible, then the bank will choose projects with negative externalities, Ω^*S*^.

#### Lemma 3

If the shareholders are socially responsible, then the effect of *e*_*M*_ on *e*_*S*_ depends on the value of (Ω^*S*^ − Ω^*M*^). If Ω^*S*^ − Ω^*M*^ > 0, then according to the formula used in Equation (11), $e_{S}^{*}$ will decrease, that is, when the negative externality of the project selected by the shareholders is greater than the project selected by the manager, then the attractiveness of project *i*_*S*_ to the shareholders will decrease. According to the formula expressed in Equation (9), $e_{M}^{*}$ will increase as $e_{S}^{*}$ decreases; it is likely that the final implementation is the one that is selected by the manager, and the negative externality Ω^*M*^ is smaller. Similarly, when Ω^*S*^ − Ω^*M*^ < 0, $e_{S}^{*}$ will increase, and $e_{M}^{*}$ will decrease. At this time, the bank's most likely choice is project, *i*_*S*_, and its negative externality Ω^*S*^ is smaller. Therefore, socially responsible shareholders will always choose projects with lower negative externalities, even if there are no CSR activists.

It is important to point out that, from the formulas expressed in Equations (9) and (11), the effects of the probability, *p*, of the CSR activists on $e_{S}^{*}$ and $e_{M}^{*}$ are ambiguous or indirect. In addition, we can extract the key factors such as $e_{M}^{*}$ and γ_*S*_ from the two stages of the game.

## Conclusion

Based on the stakeholder governance theory, this study uses a game model to analyze the game choices of shareholders, managers, and social responsibility stakeholders. There are three questions to be considered as starting points for the research.

*Question 1*: Do social responsibility stakeholders need to be included in the corporate governance structure?

From the results of Assumption 1, it can be seen that pressure from social responsibility stakeholders can effectively change the project decision of the rational board. This is similar to the findings of Sakawa and Watanabel (2020). They believed that stakeholder participation can improve corporate governance structures, and in large group companies, their opinions can significantly influence corporate operating policies. There is a gap regarding the available studies and literature. This study not only found this effect well but also measured the range of stakeholder engagement intensity, $a_{2}^{j}>\lambda \geq a_{1}^{j}$. At the same time, while considering how to minimize the costs of the social responsibility stakeholders, we proposed the optimal compression strength $\lambda =1-\left(\Pi^{A}\text{-}C_{S}\right)/\left(\Pi^{j}\text{-}\gamma_{S}\Omega^{j}\right)\equiv a_{1}^{j}$. Therefore, considering the stakeholder participation in the corporate governance structure has a positive effect.

*Question 2:* If the behavior of the social responsibility stakeholders is excessive, will it affect the bank's operating efficiency?

From the game results of the first stage, it can be seen that the project that *A* prefers can only be executed when *A* is willing to apply pressure and when *S* is willing to comprise according to those pressures. If the strength of *A* is too large, the resulting cost (*c*_*A*_(λ)) will be greater than the negative externality (Ω^*j*^) of choosing other projects. Therefore, the rational social responsibility stakeholders will not affect the performance of the bank because of an excessive behavior. Conversely, they must not be too weak; otherwise, their right to speak will become invalid. If $\lambda <a_{1}^{j}$, then the shareholders will not change their intentions. This conclusion is similar the conclusions from Renneboog et al. (2008) and Bacq and Aguilera (2021). They held that the stakeholder engagement is positively correlated with the corporate governance effectiveness, but if the engagement is too weak, the effect will be insignificant.

*Question 3:* Can shareholders' sense of CSR play a role in banks' social responsibility?

Through Assumption 2, the effort levels of shareholders and managers are negatively correlated. The reverse effect of the shareholders monitoring the manager's efforts has been demonstrated by study (Salehi and Alkhyyoon, 2021). They found when shareholders are overly aggressive, the managers may feel that their abilities are not trusted and will not put effort into fulfilling their responsibilities. This study discussed two decision-making behaviors to determine whether the shareholders have social responsibility awareness. We found that the shareholders without social responsibility will work harder to realize their preferred project *i*_*s*_, unless the probability (*p*) of social responsibility stakeholders is large enough. Through Assumption 3, we found that in any case, even if the social responsibility stakeholders are not present, socially responsible shareholders will adjust their own efforts and revise the decision-making direction in favor of the projects with lower negative externalities. This shows that the rational and socially responsible shareholders will self-consciously choose sustainable development projects. This conclusion is similar to the finding of Abeysekera and Fernando (2020). In addition, some studies suggest that the socially apathetic shareholders have a tendency to “impersonate” the socially responsible shareholders and pretend to care about the negative externalities of stakeholders (Flammer, 2013). Esker (2021) also believed that in reality, shareholders may behave socially responsible even if they do not actually care about the stakeholders. For example, this may be demonstrated by explicitly stating that the shareholders are concerned about the negative externalities and will act accordingly in the company's articles of association. The shareholders will show willingness to assume social responsibilities under the power of stakeholders or the public.

Based on the above conclusions, the participation of stakeholder representatives and the establishment of the social responsibility function of the board of directors can help promote the banks' social responsibility performance.

## Discussion

Around the world, the issue of social responsibility has attracted the attention of a number of commercial banks. For example, the Industrial and Commercial Bank of China, Bank of America, Mitsubishi UFJ Financial Group, HSBC Holdings, etc. have all published their CSR reports on a regular basis. The banking supervisory authorities of various countries have also begun to emphasize the necessity and importance the stakeholder participation in the corporate governance structure of banks to strengthen their social responsibilities. Most banks adopt a parallel two-tier governance structure model, that is, the board of directors and the board of supervisors coexist in a decentralized way. They do keep checks and balances with each other to better ensure the maximization of shareholder interests. This kind of unilateral shareholder governance structure has always only reflected the interests of individual stakeholders. The lending relationship between creditors and commercial banks, the transaction relationship between customers and banks, and the employment relationship between employees and banks are not included in the institutional arrangements for bank governance, which may lead to negative externalities to some stakeholders. Especially in the context of the COVID-19 epidemic and the ever-increasing global financial risks, it is particularly important for banks to actively undertake social responsibilities.

However, as mentioned earlier, the banks are special compared to other businesses and organizations. For example, the banking industry has a high degree of information asymmetry, strong regulation will limit the effect of shareholding constraints on banks, and the particularity of the capital structure of banks further weakens the external supervision of creditor rights. Therefore, it is particularly applicable and necessary to introduce stakeholder participation in the corporate governance structure of banks and to improve the social responsibility attribute of banks from the inside. Based on the answers to the three research questions and the conclusions drawn from this research, we have made several suggestions that can improve the social responsibility level of banks. At the same time, we also discussed the contributions and limitations of this research.

### Establish a stakeholder governance mechanism of “participation and dialogue”

Since we have come to the conclusion that stakeholder governance can improve the social responsibility level of banks, the stakeholders need to be considered in the governance structure of banks. However, there arises a question, “What kind of stakeholders should be included?” As mentioned above, this study is more inclined to governing key stakeholders than governing all stakeholders. “Participation” means that key stakeholders, as representatives of all of the stakeholders, can actually participate in or oversee the bank's decision-making process. A “dialogue” means that the key stakeholders need to engage in regular communication with all stakeholders to keep their interests abreast and to reflect on them while they participate in bank governance activities. For example, ABN AMRO Bank conducted in-depth discussions with more than 10 energy customers around the world to determine relevant draft policies. At the same time, ABN AMRO Bank maintains close contact with NGOs such as Greenpeace, the WWF, and other organizations through conversations, consultations, conferences, seminars, websites, written communications, and more. This mechanism design not only can avoid the bank having publicity problems due to stakeholder participation but also can reflect the opinions of all stakeholders to the greatest extent through the supervision of interest representatives. This study constructs a specific dialogue mechanism, as shown in Table 3.

**Table 3:** Effective dialogue mechanism: steps and results.

**Steps**	**Results**
1. Ask and understand the psychology and concerns of senior management, shareholders, and stakeholders.	Fully understand the strategic intent of different stakeholder preferences.
2. Share tacit knowledge among organizations and stakeholder groups.	Improve the organizational knowledge base to build a common understanding of company history, capabilities, performance, and future.
3. Transform tacit knowledge into explicit knowledge.	Company knowledge base coding.
4. Use shared explicit knowledge to evaluate planned and contingency strategies in the strategic rationalization process.	A reality check for the existence of a common understanding of a company's history, capabilities, performance, and future.
5. Strategy formation.	Improve strategic competitive position.

### Establish a CSR function on the bank's board of directors

The results of this study show that the social responsibility of shareholders has a positive effect on the social responsibility performance of banks. It is important to have a CSR function in the boardroom. There are two ways to optimize institutional settings.

First, the social responsibility is clearly embedded in the responsibilities of institutions and in those of the members of the board of directors. For example, when overseeing the investment decisions and lending decisions made by the bank management, the portfolio committee should take social and environmental risks into account, and at the same time, they should fully recognize opportunities for the financial innovation in the field of sustainable development. They should also actively develop financial products and services that promote the social progress and the environmental protection and fulfill the social responsibilities that are specific to the banking business. Another example is that banks can set up an external supervisor system and integrate the relationship between independent directors and the board of supervisors, all of which are conducive to the internalization of social responsibility in the development strategy of commercial banks.

Second, new institutions should be established. In addition to the existing main business decision-making committee of the board of directors, an organization such as a social responsibility committee or an environmental protection committee that is responsible for supervising the bank's implementation of social responsibility and sustainable development and for making relevant recommendations should be established. A social responsibility implementation team that is composed of the management from the various departments of the group can be established to supervise the implementation, evaluation, and communication of social responsibility policies and can also serve as the information window of the bank's social responsibility committee. A corresponding performance appraisal system for the board of directors should also be established. The bank's social responsibility performance and the board of director's social responsibility performance should also be included in the assessment indicators. The bank's social responsibility performance should be evaluated annually, a good incentive and restraint mechanism should be formed, and efforts should be made to contribute to the welfare of the environment, nature, community, and society.

### Contributions and limitations

To the perspective of theoretical contribution, the stakeholder theory has been criticized for its deviation from the business purpose of “profit first” and “shareholder first.” The development of the stakeholder theory for decades has still not overturned the essence of the profit-seeking enterprise. Therefore, some scholars believed that this theory is difficult to apply and guide practice (Kaler, 2003). Based on the game principle of “shareholder first,” this article explored the situations in which a stakeholder proposal may be rejected due to the profit concerns. Through game deduction, we were pleasantly surprised to find that even if an enterprise is profit-seeking, it is possible to seriously consider the proposals of stakeholders due to the influence of factors such as goodwill. Of course, this is not limitless, but there must be a pressure inflection point, which we also calculated in the text. Therefore, this study further improved the knowledge system of stakeholder theory and expanded the boundaries and application scope of the theory. In particular, the stakeholder governance model under the condition of “shareholder first” has been improved, and the scenarios of applying this theory to practice have been improved.

To the perspective of practical contribution, this study highlighted the key role of stakeholder governance in the implementation of CSR in the banking industry. Unlike the previous studies, we drew on game theory to analyze not only the role of stakeholders but also the role of shareholders and managers in promoting the bank's social responsibility. In addition, we measured the optimal pressure exerted by the stakeholder representatives, which, in turn, derives the dynamic performance of shareholders. At the same time, we also analyzed whether the excessive behavior of stakeholders will have adverse effects on company management, which is rarely studied by the previous studies.

What's more, there is a research gap between ours and the other available studies and literature. It should be emphasized that this study constructed the game model completely based on the rational person hypothesis. This is more in line with the profit-seeking business model of commercial banks. Compared with the assumptions of the social responsibility concept in the previous theoretical research, this study is regarded different. We believe that it would make some contributions in the stakeholder governance field. Added to that, some mathematical models proposed in this study can also serve as new references for future research.

However, this study also has some limitations. The quantitative research may lead to more convincing conclusions in this area. However, we did not do that because of the data availability. This study provided the mathematical model and deduction conclusions of the research. In the next step, some samples can be selected and relevant data can be collected, and the relevant conclusions can be verified through empirical methods. In addition, our research did not fully cover all stakeholders—such as management, employees, and other internal personnel—that may influence CSR. They also sometimes affect the actual effect of the bank's fulfillment of social responsibility at the executive level. This can be considered in the future research.

## Data availability statement

The original contributions presented in the study are included in the article/supplementary material, further inquiries can be directed to the corresponding author.

## Author contributions

JA and HD were responsible for writing the initial draft of the manuscript and putting forward the main propositions. HD was responsible for further modification and improvement of the manuscript. MY was responsible for reviewing and editing the manuscript. All authors contributed to manuscript revision, read, and approved the submitted version.

## Funding

This research was funded by the National Natural Science Foundation of China (No. 72091313).

## Conflict of interest

The authors declare that the research was conducted in the absence of any commercial or financial relationships that could be construed as a potential conflict of interest.

## Publisher's Note

All claims expressed in this article are solely those of the authors and do not necessarily represent those of their affiliated organizations, or those of the publisher, the editors and the reviewers. Any product that may be evaluated in this article, or claim that may be made by its manufacturer, is not guaranteed or endorsed by the publisher.
